# Phosphate enhance recovery from wastewater by mechanism analysis and optimization of struvite settleability in fluidized bed reactor

**DOI:** 10.1038/srep32215

**Published:** 2016-08-30

**Authors:** Ci Fang, Tao Zhang, Rongfeng Jiang, Hisao Ohtake

**Affiliations:** 1 Key Laboratory of Plant-Soil Interactions of Ministry of Education, College of Resources and Environmental Sciences, China Agricultural University, Beijing 100193, China; 2 Research Institute for Phosphorus Atlas, Waseda University, Osaka 565-0871, Japan

## Abstract

Since phosphorus, a non-renewable and non-substitutable resource, has become the principal contributor and limiting factor to water eutrophication, achieving phosphorus removal and recovery from wastewater is pretty essential. Even though struvite crystallization process has been widely used for phosphate (P) recovery in wastewater treatment, its application is hampered by difficulties controlling small particle size and crystal growth. This study was conducted to control the settleability of struvite by calculating and predicting the struvite-settling percentage (*P*_*s*_), which is always affected by the initial concentration of P (*C*_*P*_), solution pH (*pH*), reaction time (*t*), reaction temperature (*T*), agitation rate (*A*_*r*_), and inlet flow velocity (*v*_*f*_) of the fluidized bed reactor. The results showed that the settleability of struvite could be enhanced by increasing *T* and decreasing *pH*, *A*_*r*_, or *v*_*f*_, and would perform worse with overlong *t* or excessive *C*_*P*_. The dynamic variation process of the solution supersaturated index (SI) combined with the phase equilibrium theory and Ostwald ripening mechanism explained the above results sufficiently. The logistic model was chosen to predict the *P*_*s*_ under multi-factors, but the accuracy needs to be improved.

The input of high-concentration phosphorus wastewater leads to serious deterioration of surface water bodies, which can lead to the death of aquatic organisms and have negative effects on natural, economic, and social systems^1^,^2^. Because phosphorus, a non-renewable and non-substitutable resource, has become the principal contributor and limiting factor to water eutrophication, it is essential to remove it from wastewater and reuse it for other purposes. The struvite crystallization process, whose product MgNH_4_PO_4_·6H_2_O is considered the optimal phosphorus mineral substitution or slow-release fertilizer (containing 51.8% of P_2_O_5_ based on MgNH_4_PO_4_), has been widely used to recover phosphate (P) during wastewater treatment^3^. This process is regarded as the end step of wastewater treatment, even when multiple P treatment technologies are being employed. Following this step, more than 92% of P may have been removed from wastewater^4^,^5^,^6^,^7^. However, the struvite crystallization process is hindered by small particle size and crystal growth control difficulties. The PO_4_^3−^-P removal efficiency was found to increase from 96.3% to 97.1% following a decrease in the average diameter of particles (*D*_*a*_) from 40.0 μm to 31.7 μm^8^, and up to 98% P removal efficiency could be achieved at the expense of 53% of the crystals lossing^9^.

Investigations of particle settleability could help enhance P-recovery efficiency and ensure the quality of effluent by improving the settleability of struvite. Methods such as modifying the structure of the reactors and optimizing reaction parameters to enhance particle size or minimize loss of crystals could be considered as the methods to enhance the settleability of struvite. Le Corre *et al.*^10^ designed stainless steel meshes as an alternative to existing seeded fluid bed reactors (FBR) to trap poor-settling struvite, which led to a reduction in the amount of fine particles remaining in solution from 302.2 to 12 mg/L. Rahaman *et al.*^11^ designed four distinct zones in the FBR for extension of the settling time and achieved a phosphate removal rate of up to 90% and particles with sizes of up to 3.5 mm. The kinetic models of struvite nucleation and growth rate with relative supersaturation index (SI) were also used to control the settling process^12^. However, the settleability or SI could not be detected directly during the crystallization process, and some chemistry software, such as *PHREEQC*, could not calculate changes in the parameters with time. What’s more, many factors affect the process of struvite crystallization or SI all the time, like the initial concentration of P, the molar ratio of Mg and P, the solution pH value, coexisting ions (such as calcium ion, carbonate ion, suspended solids and heavy metal ions), the inlet flow velocity of FBR and so on. The initial concentration of P could determine if struvite could be formed because of the limited struvite solubility product constant 10^−13.36^ (at 298 K and standard atmospheric pressure)^13^. The molar ratio of Mg and P is 1.0 in theory, but this ratio is always over 1 for the extra consumption of other coexisting ions (like OH^−^ and CO_3_^2−^)^14^. The solution pH value influences the P forms and dominant crystal type. The crystal can be struvite only under the pH value between 7.0 and 10.0. Otherwise, Mg_3_(PO_4_)_2_ or Mg(OH)_2_ could be formed at a higher pH value^15^. The coexisting ions could reduce the size of struvite^16^, change crystal growth direction^17^, or delay the nucleation rate and the growth rate of struvite^18^. The inlet flow velocity decides the final settling velocity of crystals^19^,^20^. So the kinetic model of struvite settleability should consider these factors as many as possible.

This study was conducted to enhance P-recovery efficiency by controlling the crystallization process based on the struvite settleability in a FBR. The struvite-settling percentage (*P*_*s*_), a quantifiable index of settleability, is the key dependent variable of this study. Some quantifiable factors, such as the solution basic parameters - initial concentration of P (*C*_*P*_) and solution pH value (*pH*), the thermal parameter - reaction temperature (T), and the dynamic parameters - reaction time (*t*), agitation rate (*A*_*r*_) and inlet flow velocity (*v*_*f*_), were chosen to be the components of the *P*_*s*_ predicted model after their impact laws and mechanisms were analyzed based on the dynamic variation processes of SI.

## Results

### Influence of reaction time

**Figure 1:**
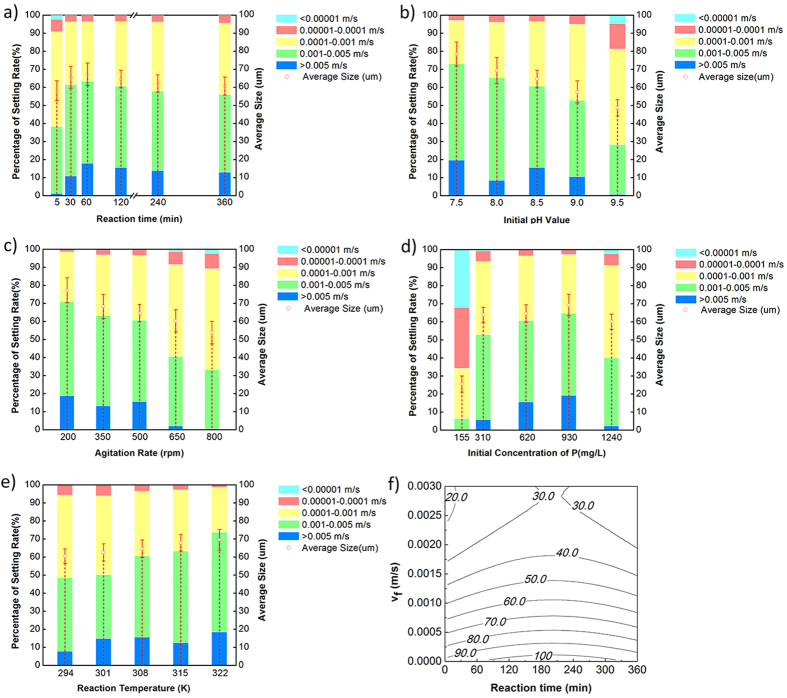
The distribution of struvite partial settling rate (**a**) with the increasing of reaction time; (**b**) in different initial pH value; (**c**) in different agitation rate; (**d**) in different initial phosphorus concentrations; (**e**) in different reaction temperatures and (**f**) changed by the inlet flow velocity with reaction time.

The settling rates of different particle sizes at different reaction times were classified into five gradients (from<1 × 10^−5^ m/s to >5 × 10^−3^ m/s), and the percentages of each gradient were calculated. Figure 1a shows the distribution of struvite particle settling rates and the variation of *D*_*a*_ with increased reaction time. As the reaction time increased, the percentage of struvite particles with a faster settling rate (over 1 × 10^−3^ m/s) initially increased, and then became stable.

In the first 5 min of the reaction, struvite crystals were produced and grown rapidly until *D*_*a*_ reached 58.141 ± 5.521 μm. During this period, particles with a settling rate >1 × 10^−3^ m/s (*P*_(*v*>0.001 m/s)_) accounted for 38.28%. Later, struvite crystals were still growing, but the growth rate slowed down gradually. Compared with the *D*_*a*_ at 5 min, the growth rate of the *D*_*a*_ at 30 min and 1 h were 15.77% and 5.64% respectively, and the *P*_(*v*>0.001 m/s)_ increased by 60.82% and 2.95%, respectively. However, when the reaction time increased from 1 h to 6 h, the *D*_*a*_ was reduced from 67.307 ± 6.211 μm to 60.722 ± 4.993 μm. The *P*_(*v*>0.001 m/s)_ also decreased, although not significantly. These findings indicate that struvite growth became stable, even with a trend of dissolution after 1 h.

### Influence of initial pH value

The initial pH of the solution determines the content of PO_4_^3−^ in the solution and therefore further affects the initial SI, ionic activity, and entire speed of struvite nucleation and growth. As shown in Fig. 1b, variation in the initial pH value was strongly negatively correlated with struvite settleability. As the pH increased, both *D*_*a*_and *P*_(*v*>0.001 m/s)_ were gradually reduced. When the initial pH was 7.5, the *D*_*a*_ of struvite reached 78.499 ± 6.646 μm, and the *P*_(*v*>0.001 m/s)_ accounted for 73.05%. However, when the pH increased to 9.5, the *D*_*a*_decreased to 48.674 ± 4.529 μm and only 28.20% of the *P*_(*v*>0.001 m/s)_ was left. The increase of S/V with the increase in initial pH value (Figure S1) meant a greater total volume of struvite particles was generated. When the initial pH was 9.5, the S/V increased greatly, resulting in production of more struvite particles than under other pH values. Moreover, the smallest *D*_*a*_ was observed at pH 9.5, indicating that the largest number of struvite particles were generated under this pH. In contrast, when the pH was 7.5, the number of struvite particles was the least, but the *D*_*a*_ was the largest. These results were consistent with those of previous studies^21^,^22^.

### Influence of agitation rate

Agitation rate is an important parameter influencing the inter-solute mass transfer effect and the width of the solution metastable zone. As shown in Fig. 1c, there was a negative correlation between agitation rate and struvite settling rate. Specifically, at a higher agitation rate, the *D*_*a*_and *P*_(*v*>0.001 m/s)_ values were lower. *D*_*a*_was the largest when the agitation rate was 200 rpm, reaching 77.301 ± 6.908 μm, while *P*_(*v*>0.001 m/s)_ reached 70.91%. However, as the agitation rate increased gradually, bigger particles were reduced gradually and the *D*_*a*_and *P*_(*v*>0.001 m/s)_ eventually fell to 33.16% and 53.837 ± 6.238 μm respectively at an agitation rate of 800 rpm. As shown in Figure S2, the total volume of struvite particles in the unit volume of solution increased significantly as the agitation rate increased, especially above 650 rpm. According to the conclusion that the particle size decreased with the increase of agitation rate, it could be determined that struvite particles had a large quantity but small size with a faster agitation rate, while they had a small quantity but large size with a slower agitation rate, similar to the case with initial pH.

### Influence of initial concentration of phosphorus

The initial concentration of P (*C*_*P*_) directly influences the initial SI of the solution, thus affects struvite nucleation and growth. Five *C*_*P*_ gradients of simulation wastewater were prepared for struvite crystallization. Here is the percentage distribution of struvite settling rate at different *C*_*P*_ in Fig. 1d. The tendency of the *D*_*a*_ and the *P*_(*v*>0.001 m/s)_ initially increased, then decreased as *C*_*P*_ increased. When the C_P_ was 155 mg/L, the settling rate of struvite particles was mainly concentrated at 1 × 10^−5^ –1 × 10^−3^ m/s and the *D*_*a*_ was 20.567 ± 4.457 μm, while the settling rate and the *D*_*a*_at *C*_*P*_ values over 155 mg/L were mainly concentrated at 1 × 10^−4^ –5 × 10^−3^ m/s and 60–70 μm, respectively. The maximum *D*_*a*_ and the *P*_(*v*>0.001 m/s)_ reached 69.333 ± 5.958 μm and 64.70% respectively at the *C*_*P*_ 930 mg/L, and then respectively reduced to 60.092 ± 4.993 μm and 40.04% at *C*_*P*_ 1220 mg/L.

### Influence of reaction temperature

Reaction temperature has a significant effect on thermodynamic parameters such as SI and solubility products of crystals. The reaction temperatures were set as 294 K, 301 K, 308 K, 315 K, and 322 K, respectively. Figure 1e shows that as reaction temperature increased, the *D*_*a*_ increased gradually, as did the *P*_(*v*>0.001 m/s)_. When reaction temperature was 294 K, the *D*_*a*_was 60.567 ± 5.764 μm and the *P*_(*v*>0.001 m/s)_ accounted for 48.61%. As temperature increased, particles with higher settling rates increased gradually to a maximum of 322 K, while the *D*_*a*_ increased to 69.534 ± 3.929 μm and the *P*_(*v*>0.001 m/s)_ increased to 73.72%. Although the increase of *D*_*a*_was not significant, the increase of P_(*v*>0.001 m/s)_ was significant and the homogeneity of struvite particles improved.

### Influence of inlet flow velocity

Struvite crystallization generally occurs in a FBR, and its inlet flow velocity (*v*_*f*_) directly influences the settling rate of struvite. Six gradients of *v*_*f*_ were assumed (3 × 10^−5^ –3 × 10^−3^ m/s) according to the reference data^12^,^19^,^20^,^23^. Figure 1f presents the contour map of changes in the settling percentage in response to the *v*_*f*_ with reaction time. The settling rate of struvite decreased as the *v*_*f*_ and reaction time increased, while the degree of the settling rate tended to be smaller. When the *v*_*f*_ was less than 1 × 10^−4^ m/s, the settling rate of struvite could reach above 90%. However, when it was over 2.8 × 10^−3^ m/s, two valleys appeared in Fig. 1f. This might be attributed to the fact that when the *v*_*f*_ rose to a certain value, the turbulence of the solution could promote the solid-liquid mass transfer at the beginning, but also enhance the effects of wear between crystals later, which led to the overall small particle size and lower settling rate^24^,^25^.

### Quantitative investigation of struvite-settling percentage

To enable prediction of the struvite-settling percentage, polynomial model (Eq. S2) and logistic model were considered. Compared the astringency, statistically significance and prediction error of two models (Table S1), the logistic model was chosen as a better one and the basic formula was as follows:


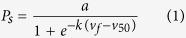


where *P*_*s*_ is the struvite-settling percentage (%); *a* is the extreme value of *P*_*s*_, as the value of *P*_*s*_ is between 0 and 100 (%), so the value of *a* is 100 (%); −*k* is a rate parameter with a higher −*k* indicating a slower descent speed of the ratio; and *v*_*50*_ is the velocity of up-flow fluid, where *P*_*s*_ is 50%. What’s more, the model assumes no impurities effect and equimolar concentrations of N and P in the fluid.

Principal component analysis (PCA) was used to establish the models of parameters (−*k* and *v*_*50*_) in the logistic model influenced by multiple factors. The density and viscosity of fluid, as essential parameters of SI, were added in the model. Based on the experimental data (Table S2), Eqs S4 and S6 show the prediction models of fluid density and fluid viscosity^26^, respectively. Finally, the models of struvite −*k* and *v*_*50*_ were established by the least-squares method and the formulas were as follows:


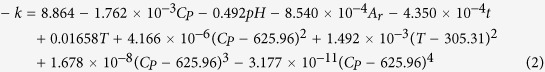



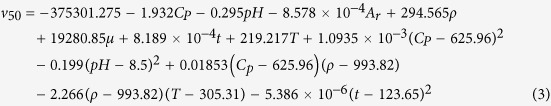


where, *C*_*P*_ is the initial concentration of P (mg/L), *pH* is the initial pH value, *A*_*r*_ is the agitation rate (rpm), *t* is the reaction time (min) and *T* is the reaction temperature (K). Analysis of variance was further applied to evaluate the significance and adequacy of the models and identify the complex relationship between factors and parameters. The results are summarized in Table 1. Based on the high correlation coefficients (R^2^ = 0.9661 and 0.9738) and very low Prob. >|*t*| values for all factors as presented in Table 1, these predicted models have good fitness and could be used to predict the struvite-settling percentage with multiple factors.

**Figure 2:**
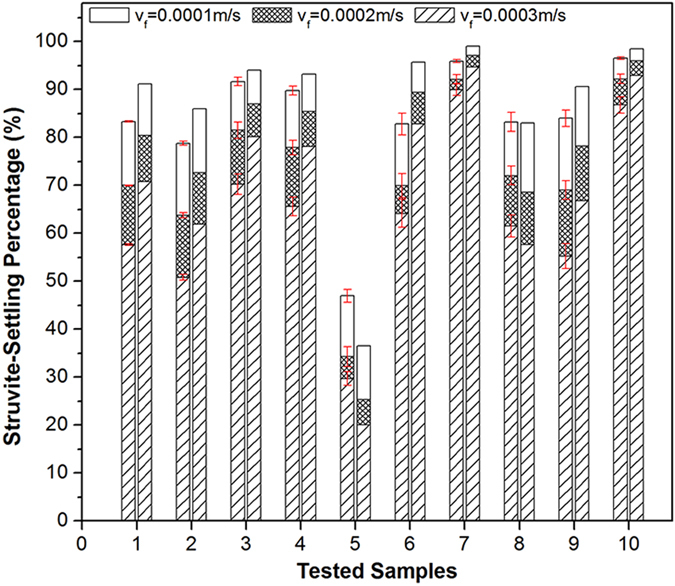
The contrast of simulation values and measured values of struvite-settling percentage.

Ten tests (in random) were used to test the accuracy of the model (Table S3). The settling rates were tested under *v*_*f*_ values of 0.0001 m/s, 0.0002 m/s, and 0.0003 m/s, respectively, and matched in models at the same time. The comparison results are shown in Fig. 2. The predicted data were always higher than the experimental data. Even though there were some differences between the tested and matched results in these 10 groups, there were no significant differences based on analysis of variance. Therefore, this model could be used to predict the basic struvite particles settling rate, but the accuracy still needs to be improved.

## Discussion

**Figure 3:**
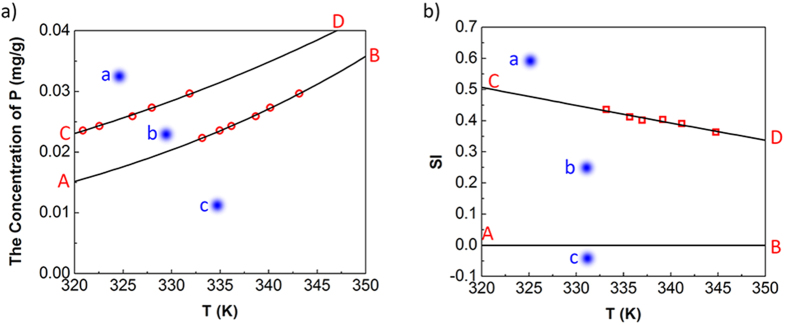
(**a**) the metastable zone of struvite; (**b**) the relationship between the supersaturation index (SI) of struvite solution and struvite crystallization.

The metastable zone of struvite under different conditions and the dynamic variation process of SI were tested to further verify the results. The mechanisms of struvite settleability were explained based on the theory of phase balance. Figure 3 shows the relationship of fluid supersaturation and the struvite crystallization. Figure 3a shows the traditional expression way of metastable zone. For a better comparison, change the concentration of P for SI and the relationship between the degree of supersaturation of struvite solution and struvite crystallization was shown in Fig. 3b. In both of two figures, line AB represents the solubility curve of struvite and line CD represents the supersaturation curve. Point *a* is in the unstable supersaturated area above line CD (SI > line CD), which is favorable for crystal nucleation. Point *b* is in the metastable supersaturated area between line AB and line CD (0 < SI < line CD), which is favorable for the growth of crystals. Point *c* is in the unsaturated stable area below line AB (SI < 0), which is not favorable for the nucleation of crystals, or is favorable for the dissolution of crystals.

**Figure 4:**
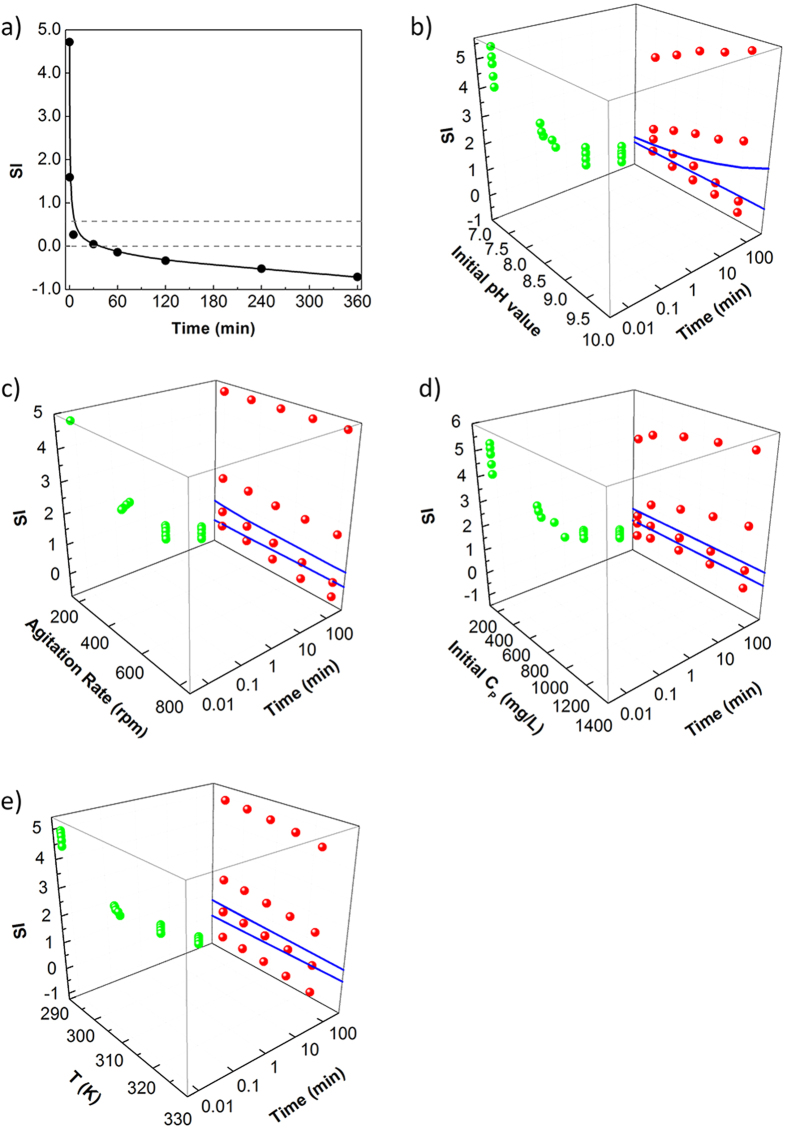
The variation of SI with the changes of (**a**) the reaction time (**b**) initial pH value and time (**c**) agitation rate and time (**d**) initial concentration of P and time (**e**) reaction temperature and time.

Figure 4 shows the dynamic variation process of SI for different factors. The area between the two dotted lines is the metastable zone. At the beginning of the reaction, all of the SI values were higher than the SI required for struvite crystallization, so a large amount of NH_4_^+^, PO_4_^3−^ and Mg^2+^ were used to form struvite particles, and the mass diffusion transfer between the solution and crystals played a dominant role. The SI under different conditions dropped sharply after nucleation, but remained higher than the metastable zone, except at 155 mg/L of C_P_ (Fig. 4d), and the crystals were still mainly in the nucleation process. Later, the SI value began to drop to the metastable zone. Crystals were in the growth stage, rapidly growing to form crystal lattices by absorbing NH_4_^+^, PO_4_^3−^ and Mg^2+^ in the solution on the crystal lattices own surface and polymerizing with other crystals to form larger crystals. The smallest crystals might be dissolved and particle aggregation/re-crystallization phenomena may occur^27^. However, as the drop of the pH and the concentrations of NH_4_^+^, PO_4_^3−^ and Mg^2+^, the SI further decreased. Therefore, the mass transfer effect weakened, the growth rate of crystals decreased, and particles with high settling rates were reduced^28^. The retention time for the SI value remaining in the metastable zone was the key factor determining the settleability of struvite. Even though some other factors (initial pH value, agitation rate, and temperature) could also influence the width of the metastable zone, a longer retention time was associated with better settleability of struvite. Once the SI was less than 0, the growth of crystals became restricted, the ion transfer effect from crystals to liquid phase was enhanced, the surface polymerization force of crystals was reduced, and the crystals started to dissolve. Therefore, the longer reaction time might further deteriorate the settleability of struvite (Fig. 4a).

As shown in Fig. 4b, even though a higher pH value could widen the metastable zone, more crystal nuclei were produced under a higher pH value, resulting in a quicker decrease of SI^29^. Le Corre *et al.*^30^ tested the decrease of the negative zeta-potential (from −17.5 ± 1.1 to −23.1 ± 1.4 mV) of the solution when the pH increased from 8.5 to 9.5 and indicated that although it did not link the values to particle size directly, it was likely to have an effect on crystal growth kinetics and lead to eventual destabilization of particles by aggregation. As a result, it might take quite a long time for crystals to nucleate at a lower initial pH value and a lower initial SI, but once crystal nuclei were produced, a longer time is required for growth. Even though they all had a negative impact on zeta potential, the overall settleability at a lower initial pH value was better than that under a high initial pH value.

The same principles could be used to explain the influence of agitation rate. Even though a faster agitation rate could improve mass transfer in the process of crystallization and shorten the time of crystal nucleation^24^,^31^, it resulted in a thinner boundary layer between the solid phase and liquid phase (Fig. 4c). On the contrary, a slower agitation rate could widen the metastable zone of struvite so that crystals had a longer time for growth. Therefore, as shown by Cerrillo *et al.*^32^, widening of the metastable zone was the main reason for the better settleability at a slow agitation rate.

The reason why the settleability decreased after the *C*_*P*_ was over 930 mg/L (Fig. 1d) could be explained by the data shown in Fig. 4d. At 5 min, the solution SI was basically at the metastable zone, except for 155 mg/L, and SI increased first, reaching a maximum value at 930 mg/L, then decreasing. At 2 h, all of the SIs were less than 0 and the variation trend was similar to that at 5 min. This was because the increase of *C*_*P*_ would increase the concentration of P, but decrease the ion activity coefficient. Both parameters led to the inevitable extremum of the free ionic activity product (Eq. 14), and then led to SI reaching the maximum value at 930 mg/L. So calculation of the initial SI using the *PHREEQC* software could not determine the final settleability of struvite^33^,^34^. Ye *et al.*^22^ tested changes in the negative zeta potential of the solution with increasing *C*_*P*_ and found that the negative charge of the solution would decrease first, then increase. The smaller the negative charge was, the stronger the struvite polymerizing potential was. Their results were similar to those of this study and illustrated that, in a certain range of *C*_*P*_, the settleability of struvite would be better as *C*_*P*_ increased, while a much higher *C*_*P*_ promoted the nucleation of crystals, but reduced the polymerizing potential of struvite, shortened the effective time for crystals growth, and in turn reduced the *D*_*a*_ and decreased the settleability of crystals.

When compared with the results shown in Fig. 1e, the variation of SI with temperature and time followed an opposite trend (Fig. 4e). At the beginning of the reaction, the solution SI decreased with increasing temperature. Therefore, it was difficult to nucleate at higher temperature and the nucleation time was longer. What’s more, the SI at 322 K decreased to less than 0 quickly at 5 min, making it more difficult for crystals to grow. However, the settleability of crystals didn’t keep deteriorating as temperature increases. These conflicting conclusions showed that the single solid-liquid phase equilibrium theory could not explain the influence of temperature on struvite settleability. Both Ronteltap *et al.*^21^ and Ariyanto *et al.*^29^ showed that the rise of temperature could increase the solubility products of struvite and aggravate the difficulty of nucleation, but could also accelerate the growth rate of struvite. When the reaction temperature rose from 283 K to 303 K, the *D*_*a*_ increased by 53.8%, and the growth rate increased from 2.5 × 10^−9^ m/s to 5.2 × 10^−9^ m/s. This might have occurred because at higher temperatures the clusters adsorbed onto the crystal surface were more active, causing a higher surface-integration rate, which was significantly stronger than the solid-liquid mass transfer effect so that crystals had a tendency to increase. The Ostwald ripening mechanism also pointed out that a higher temperature could dissolve micro crystals, promote secondary nucleation of larger crystal nuclei, and as a result, increase the particle size of crystals^35^. In short, variations in reaction temperature had a bidirectional effect on the growth of struvite crystals, with a negative effect caused by the decrease of SI, and a positive effect caused by the increase of surface activity of crystals. In this study, the increase of surface activity of crystals was caused by the increase of temperature, promoting the surface polymerization of crystals, which was a major control step for the growth of crystals.

## Methods

### Materials

Batch experiments of struvite precipitation reaction were carried out using 12 hydration disodium hydrogen phosphate (Na_2_HPO_4_·12H_2_O), ammonium chloride (NH_4_Cl), and magnesium chloride hexahydrate (MgCl_2_·6H_2_O). The stock solutions of Na_2_HPO_4_·12H_2_O, NH_4_Cl and MgCl_2_·6H_2_O, as HPO_4_^2−^, NH_4_^+^ and Mg^2+^ providers, respectively, were made by dissolving crystals in deionized water. Synthetic wastewater was made by mixing equimolar concentrations and equal volumes of Na_2_HPO_4_·12H_2_O and NH_4_Cl solutions, and the initial P concentration varied from 155 mg/L to 1240 mg/L. The synthetic wastewater and MgCl_2_·6H_2_O were stored separately in a refrigerator (277 ± 2 K). The initial solution pH was adjusted to 7.5–9.5 using 0.1 M sodium hydroxide (NaOH) solution and 0.1 M hydrochloric acid (HCl) solution.

### Main experiments

**Figure 5:**
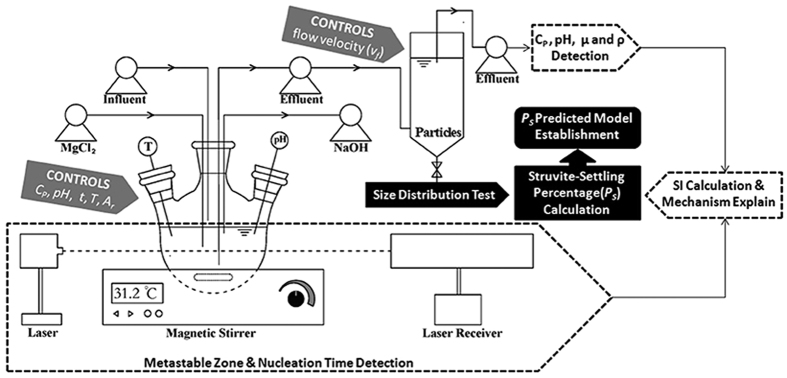
Schematic illustration of the main experiments.

A laboratory-scale FBR setup and the workflow of the main experiments are shown in Fig. 5. A 500-mL three-necked, round-bottomed flask was used for crystallization. This flask was placed in a temperature-controlled magnetism mixer (SZCL-2A, Henan, China) and mixed using a magnet with a length of 30 mm and a diameter of 3 mm. Next, 300 mL of synthesis wastewater was put into the flask at a specified temperature (294 K–322 K) and mixed at a specified speed (200 rpm–800 rpm). An equal molar concentration of MgCl_2_·6H_2_O solution was then added into the flask at a constant rate using a peristaltic pump (BT100-2, Shanghai, China) and the timer started at the same time. The He-Ne laser (633 nm, GY-11, Tianjin, China) was used to determine the time of crystal formation with the sudden increase of the laser received signal. A certain volume of struvite turbid liquid was removed on a constant height to the sedimental tube at a specified time (from 5 min to 6 h) to determine the size distribution by laser granulometry (Mastersizer 2000, Worcestershire, UK).

### Metastable region measurement

A total of 300 mL synthesis wastewater was put into the flask at 293 K and mixed at 500 rpm, after which a specified volume (0–5.00 mL) of an equal molar concentration of MgCl_2_·6H_2_O solution was added into the flask. After the crystal formed, dissolution of the crystal with increasing temperature was measured by the He-Ne laser. The temperature (*T*_1_, K) was recorded along with the crystal dissolution and disappearance. The temperature was then increased by 5 K and held for 1 h. The temperature was then decreased at the same rate and the level (*T*_2_, K) at which the numerical reading of the laser increased suddenly was recorded. The metastable zone (*∆T*, K) of struvite could then be measured using the following formula:


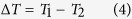


### Calculation of settling velocity

In fluid treatment, the equations of particle settling in different flow regimes were determined as follows:


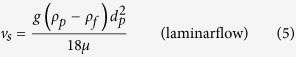



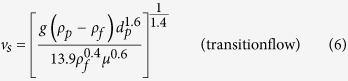



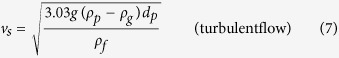


where *g* is the acceleration owing to gravity, 9.81 m/s^2^; *ρ*_*p*_ and *ρ*_*f*_ are the density of particle and fluid, respectively, kg/m^3^; *d*_*p*_ is the average diameter of particles, m; *μ* is the dynamic viscosity of the fluid, Pa·s. However, struvite is composed of rhombic crystals, which have an irregular shape. Therefore, the particle settling was amended for the fractals, and the equation of the correction factor *P*_*s*_ becomes^36^:


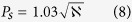


where χ is the sphericity factor, which can be calculated by the percentage of the same volume sphere surface area and the particle surface area. Therefore, the correction factor *P*_*s*_ could also be described by the expression:


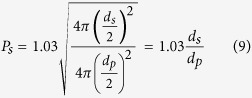


where *d*_*s*_ is the diameter of the same volume sphere, m. Accordingly, the terminal settling velocity of struvite can be expressed as follows:


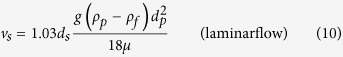



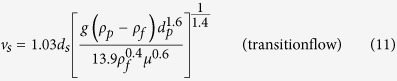



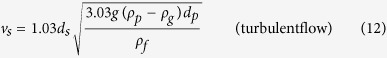


### Calculation of saturation index

The saturation index (SI) was calculated to determine supersaturation of the precipitate phase in the solution using the following equation:


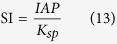


where *IAP* is the free ionic activity product and *K*_*sp*_ is the thermodynamic solubility product constant. *IAP* and *K*_*sp*_ can be calculated by the formula:


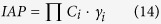



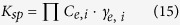


where *C*_*i*_ and *γ*_*i*_ are the concentration and ion activity coefficient of ion *i* in the solution and *C*_*e,i*_ and *γ*_*e,i*_ are the concentration needed for saturation and ion activity coefficient at that time of ion *i*. The ionic activity coefficient (*γ*) can be calculated by Davies equation^37^, which is the modification of the Debye–Hückel equation^38^:


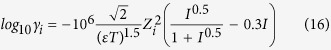


where *ε* is the dielectric constant and may be determined using the Eq. S7^39^; *Z*_*i*_ is the number of replaceable hydrogen atoms or their equivalent; and *I* is the ionic strength of solution, mol/L, which can be determined by Eq. S8^40^. The Davies equation (Eq. 16) is typically in error by 1.5% and 5–10% at ionic strengths between 0.1 and 0.5 M, respectively^41^.

## Additional Information

**How to cite this article**: Fang, C. *et al.* Phosphate enhance recovery from wastewater by mechanism analysis and optimization of struvite settleability in fluidized bed reactor. *Sci. Rep.* **6**, 32215; doi: 10.1038/srep32215 (2016).

## Supplementary Material

Supplementary Information

## Figures and Tables

**Table 1:** ANOVA for struvite-settling percentage predicted model.

−*k*				*v*_50_			
Process Parameters	Prob. >| *t* |^a^	Process Parameters	Prob. >| *t* |	Process Parameters	Prob. >| *t* |	Process Parameters	Prob. >| *t* |
R^2^	0.9661	*A*_*r*_^2^	0.0421(+)	R^2^	0.9738	*t*	0.0426(+)
*C*_*p*_	0.0002(−)	*T*^ 2^	0.0002(+)	*C*_*p*_	0.0001(−)	*C*_*p*_^2^	0.0002(+)
*pH*	<0.0001(−)	*C*_*p*_^3^	<0.0001(+)	*pH*	<0.0001(−)	*pH*^ 2^	0.0096(−)
*A*_*r*_	0.0006(−)	*C*_*p*_^4^	<0.0001(−)	*A*_*r*_	0.0002(−)	*C*_*p*_ × *ρ*	<0.0001(+)
T	0.0001(+)			*ρ*	0.0001(+)	*ρ*^2^	<0.0001(−)
*t*	0.1730(−)			T	0.0002(+)	*ρ* × *T*	<0.0001(−)
*C*_*p*_^2^	0.0026(+)			*μ*	0.0002(+)	*t*^2^	0.0285(−)

^a^A probability t value (“Prob. > |t|”) less than 0.05 indicates that the parameter has a significant meaning to the model. ^b^(+ ) indicates a positive influence and (−) a negative influence on the struvite-settling percentage.
