# Supplementary material for: Phosphate enhance recovery from wastewater by mechanism analysis and optimization of struvite settleability in fluidized bed reactor
Source: Sci Rep. 2016 Aug 30;6:32215. doi: 10.1038/srep32215 (PMC5004189; doi:10.1038/srep32215)
Supplement: Supplementary Information [file srep32215-s1.pdf]

Supplementary Information:

Phosphate enhance recovery from wastewater by mechanism analysis and optimization of struvite settleability in fluidized bed reactor

Ci Fang[1], Tao Zhang[1,*], Rongfeng Jiang[1], Hisao Ohtake[2]

[1]Key Laboratory of Plant-Soil Interactions of Ministry of Education, College of Resources and Environmental Sciences, China Agricultural University, Beijing 100193, China

[2]Research Institute for Phosphorus Atlas, Waseda University, Osaka 565-0871, Japan

[*]**Corresponding author：**

Tao Zhang

Tel：86-010-62733638

E-mail: taozhang@cau.edu.cn

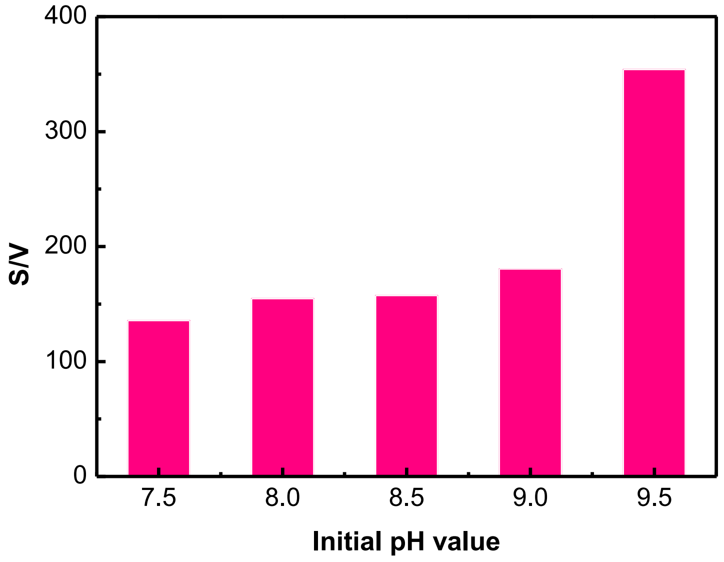

Supplementary Figure 1. Shading degree (S) per unit volume (V) of struvite turbid liquid (detected by the Mastersizer detector) at different initial pH value.

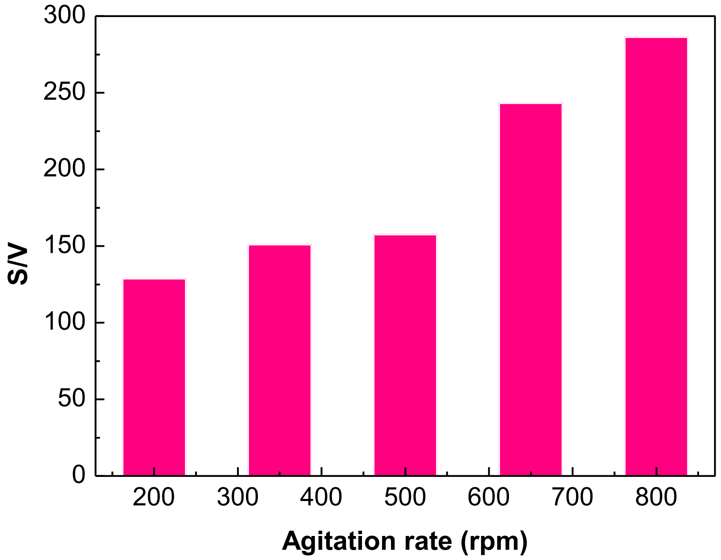

Supplementary Figure 2. Shading degree (S) per unit volume(V) of struvite turbid liquid at different agitation rate.

Polynomial model

The basic formula of polynomial model is as follows:

$$P_S = C + a_1X_1 + a_2X_2 + a_3X_3 + \ldots + a_nX_n + b_1(X_1-c_1)^2 + b_2(X_2-c_2)^2 + b_3(X_3-c_3)^2 + \ldots + b_n(X_n-c_n)^2 \quad \text{(S1)}$$

where X_1 to X_n represent the impact factors, such as initial concentration of phosphate, pH value and so on; the C, a_1 to a_n and b_1 to b_n are constants of constant term, the first degree term and quadratic term, respectively.

Finally, the model was established by the least-squares method and the formula was as follows:

$$P_s = -37160.45 - 3244.01v_f - 14.66pH - 0.1439C_P - 0.04444\mathrm{A_r} + 26.35T + 27.50\rho + 2423.45\mu + 0.04817t + 9.561\times10^6\times(v_f - 1.071\times10^{-3})^2 - 9.496\times(pH-8.5)^2 - 3.102\times10^{-4}\times(t-123.7)^2 \quad \text{(S2)}$$

where, v_f is the inlet flow velocity (m/s), pH is the initial pH value, C_P is the initial concentration of P (mg/L), A_r is the agitation rate (rpm), T is the reaction temperature (K) and t is the reaction time (min). Analysis of variance was further applied to evaluate the significance and adequacy of the model and identify the complex relationship between factors and parameters. The results are summarized in Table S1 and the comparison results between tested and predicted ones of ten tests in random are shown in Figure.S3. Compared the correlation coefficients (R^2=0.9435) and the astringency with logistic model, the fitness of logistic model is better than polynomial model and the struvite-settling percentage predicted by polynomial model would be out of the valid range (from 0% to 100%) under some factors. So the logistic model was a better one comparatively.

Supplementary Table 1. ANOVA for struvite-settling percentage prediction by the polynomial model

Process Parameters	Prob. > | t |[a]	Process Parameters	Prob. > | t |
R^2	0.9435	C_P	<.0001(-)
v_f	<.0001(-)[b]	pH	<.0001(-)
v_f^2	<.0001(+)	A_r	<.0001(-)
ρ	<.0001(+)	pH^2	<.0001(-)
T	<.0001(+)	t^2	<.0001(-)
μ	<.0001(+)	t	0.0002(+)

a A probability t value ("Prob. > | t |") less than 0.05 indicates that the parameter has a significant meaning to the model.

b (+) indicates a positive influence and (-) a negative influence on the struvite-settling percentage.

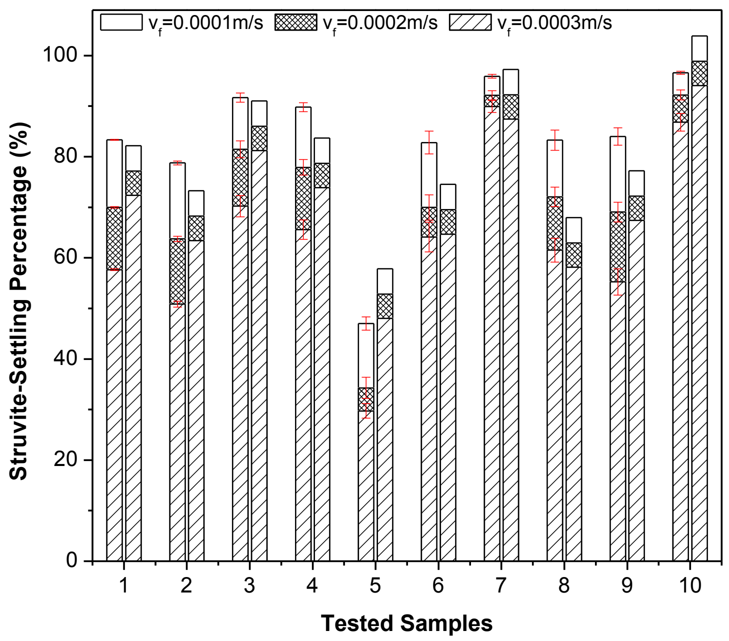

Supplementary Figure 3. The contrast of simulation values and measured values of struvite-settling percentage by the polynomial model.

Fluid density and viscosity measurements

The fluid after reactions had been collected to test the fluid density and viscosity at a specified temperature. The density of fluid was calculated by the expression:

$$\rho_f = \frac{m_f}{V_f} \quad \text{(S3)}$$

where ρ_f is the density of fluid, kg/m^3; m_f is the quality of fluid, kg; V_f is the volume of the corresponding quality, m^3. By measuring a certain volume of fluid by the transfer pipette to a beaker in the electronic balance (0.0001g precision, ME204, Shanghai, China) as soon as possible, the density of fluid could be calculated. The viscosity of fluid at a specified temperature could be measured by viscometer (NDJ-5S, Shanghai, China). All the measurements were repeated three times and the average values were calculated.

The measured densities and viscosities of the fluid at different reaction temperatures (T) and different initial phosphorus concentrations (C_P) are shown in Table S2.

Supplementary Table 2. The measured densities and viscosities of fluid at different reaction temperatures (T) and initial phosphorus concentrations (C_P)

T (K)	C_P (mg/L)	ρ_f (kg/m^3)	μ (Pa s)
294		995.41±0.29	0.972±0.007
301		994.17±0.24	0.908±0.004
308	620	993.36±0.16	0.848±0.003
315		991.95±0.24	0.793±0.008
322		988.76±0.60	0.742±0.003
	155	989.48±0.16	0.875±0.005
	310	990.37±0.57	0.853±0.004
308	620	993.36±0.16	0.848±0.003
	930	994.89±0.62	0.84±0.000
	1240	995.9±0.61	0.86±0.000

Both temperature and initial concentration of P would influence the density of fluid. Principal component analysis (PCA) and least square method were used in the SAS software to establish the prediction model of density. The response surface is shown in Figure.S4 and its expression is as

followed:

$$\rho_f = 505.24 + 3.354T + 0.01164C_P - 0.0058T^2 - 3.952 \times 10^{-6}C_P^2 \quad R^2=0.974 \quad (S4)$$

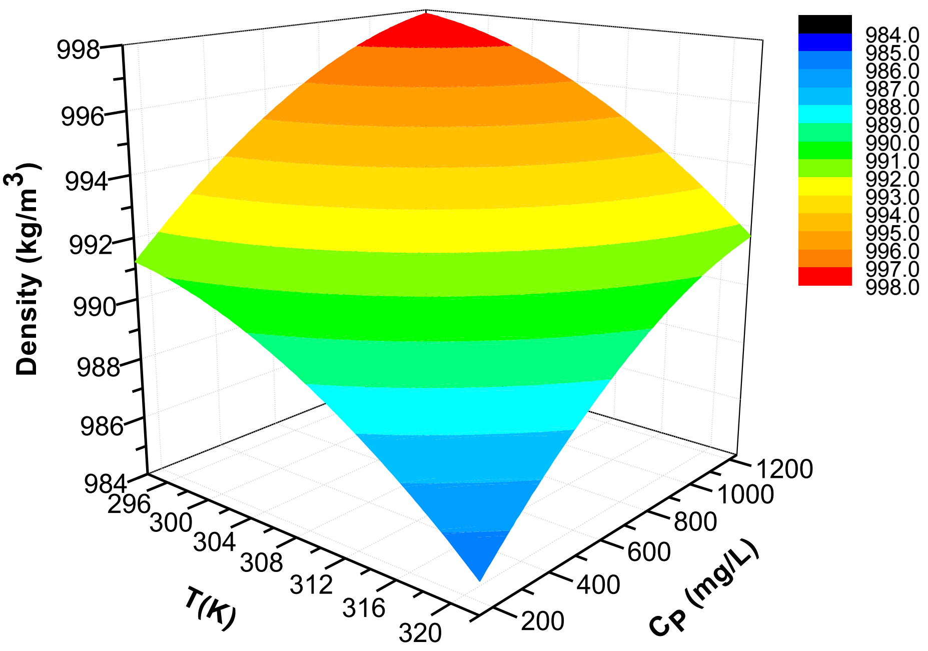

Supplementary Figure 4. The density of fluid changed by temperature and initial phosphorus concentration

Heridary et al. (2011) established an exponential model for simulating the relationship between temperature and viscosity[25]. The basic model becomes:

$$\mu = Ae^{\frac{B}{T}} \quad (S5)$$

where, *A* and *B* are constant. So the prediction model of fluid's viscosity could be expressed as:

$$\mu = 0.044e^{\frac{910.13}{T}} \quad R^2=0.999 \quad (S6)$$

Based on the high correlation coefficients (R^2=0.999), Eq.S6 had good fitness and could be used to predict the viscosity of fluid.

Supplementary Table 3. Parameters of samples for model validation

Samples	T (K)	C_p (mg/L)	ρ （kg/m3）	μ (mPa s)	T (min)	Ar (rpm)	pH
1	313	620	992.39	0.806	180	750	9.0
2	298	620	995.25	0.933	180	750	9.0
3	308	655	993.86	0.845	240	500	9.0
4	308	655	993.86	0.845	360	500	9.0
5	308	185	989.95	0.845	240	500	8.5
6	308	255	990.65	0.845	240	500	8.5
7	308	620	993.63	0.845	360	450	7.5
8	308	620	993.63	0.845	360	450	9.5
9	308	620	993.63	0.845	240	1100	8.5
10	308	620	993.63	0.845	240	350	8.5

The equation calculating the dielectric constant ε is expressed as follow:

$$\varepsilon \cong 78.54\{1 - [0.004579(T - 298)] + 11.9 \times 10^{-6}(T - 298)^2 + [28 \times 10^{-9(T-298)^3}]\} \quad \text{(S7)}$$

where, *T* is the absolute temperature, K.

The equation calculating he ionic strength of solution *I* (mol/L) is expressed as follow:

$$I = \frac{1}{2}\sum C_i Z_i^2 \quad \text{(S8)}$$

where, C_i stands for the concentration of ion *i* in the solution; Z_i is the number of replaceable hydrogen atoms or their equivalent.
